# Porous Polymersomes as Carriers for Silver Nanoparticles and Nanoclusters: Advantages of Compartmentalization for Antimicrobial Usage

**DOI:** 10.1021/acs.biomac.3c00925

**Published:** 2023-11-10

**Authors:** Bela B. Berking, Lucía Mallen-Huertas, Sjoerd J. Rijpkema, Daniela A. Wilson

**Affiliations:** Systems Chemistry Department, Institute for Molecules and Materials, Radboud University, Nijmegen 6500 HC, The Netherlands

## Abstract

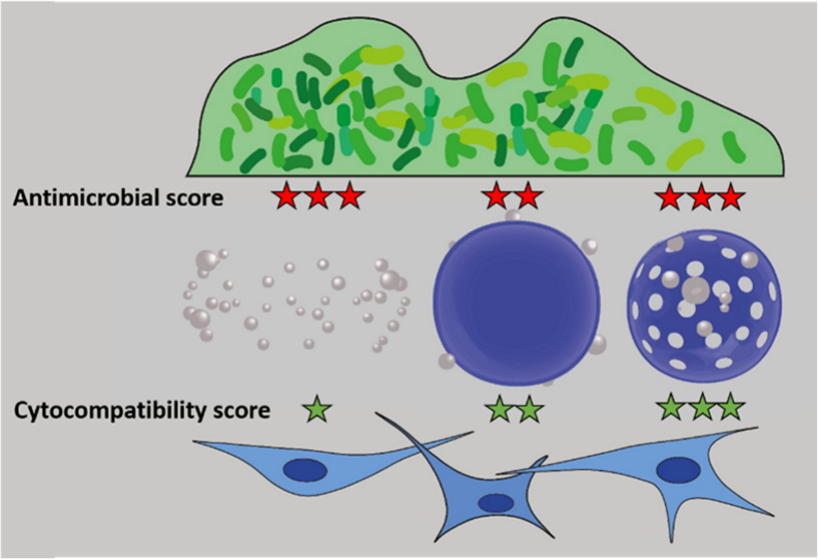

The global threat to public health posed by antibiotic-resistant bacterial infections requires the exploration of innovative approaches. Nanomaterials, particularly silver nanoparticles (AgNPs) and nanoclusters (AgNCs), have emerged as potential solutions to address the pressing issue of a bacterial healthcare crisis. However, the high cytotoxicity levels and low stability associated with AgNPs and AgNCs limit their applicability. To overcome these challenges, AgNCs and AgNPs were synthesized in the presence of porous polymersomes, resulting in a compartmentalized system that enhances stability, reduces cytotoxicity, and maintains high antimicrobial activity. The encapsulated particles exhibit a distribution of silver components on both the surface and the core, which is confirmed through the analysis of surface charge and center of mass. Moreover, our investigation demonstrates improved stability of the nanoparticles and nanoclusters upon entrapment in the porous system, as evidenced by the ion release assay. The antimicrobial effectiveness of porous polymersomes containing AgNPs and AgNCs was demonstrated by visualizing the biofilms and quantifying the penetration depth. Furthermore, cytotoxicity studies showed that compartmentalization increases cell compatibility for AgNC-based systems, showcasing the many advantages this system holds.

## Introduction

According to the World Health Organization (WHO), antibiotic resistance poses a significant threat to public health worldwide.^1^ It not only causes extended hospital stays and higher medical expenses but also increased mortality rates.^2^ Immunocompromised patients, including those with autoimmune diseases, cancer, diabetes, or HIV, are particularly vulnerable to antibiotic resistance.^3^ In fact, in 2019 alone, there were 4.95 million deaths globally associated with antimicrobial resistance (AMR) with 1.27 million directly attributable to AMR.^4^ The financial burden is concerning as well, and resistance to antibiotics is estimated to cost more than 300 billion dollars annually by 2050.^5^

This issue is aggravated by the ability of resistant pathogens to form biofilms, consortiums of microorganisms that form a protective matrix of extracellular material. This matrix, known as the extracellular matrix (ECM), consists of exopolysaccharides, extracellular DNA, and proteins.^6^ Its primary function is to provide integrity and protection against antimicrobials and the immune system. Remarkably, the ECM can prevent the penetration of positively charged antibiotics through interaction with its components.^7^ Additionally, the transfer of genes associated with antibiotic resistance occurs at a high rate.^8^ Consequently, bacteria in biofilms exhibit a 10- to 1000-fold increase in antibiotic resistance in comparison to bacteria in planktonic state.^9^

In medical settings, bacteria in biofilms have been found in living tissues and on the surface of medical devices leading to nosocomial infections that increase the risk of complications in patients with underlying conditions.^6,10^ Notably, common pathogens belong to the ESKAPE group, which includes the ubiquitous *Pseudomonas aeruginosa*, which is an opportunistic bacterium often associated with cystic fibrosis, chronic wounds, keratitis, and medical device colonization.^6^*P. aeruginosa* is responsible for 10–15% of global infections and frequently contributes to upper respiratory tract infections and catheter-associated urinary tract infections.

*P. aeruginosa* is characterized by forming biofilms that provide both structural support and drug resistance.^11^ The biofilm’s structural integrity mainly relies on extracellular proteins and exopolysaccharides. Interestingly, exopolysaccharides also play a role in antibiotic resistance. *P. aeruginosa*’s ECM consists of three main exopolysaccharides: Psl, Pel, and alginate. While Psl and Pel serve as the primary structural components of the biofilm, it has been observed that Psl interacts electrostatically with antibiotics, leading to their sequestration within the matrix.^12^ In addition, the presence of alginate contributes to the formation of mucoid biofilms, which result in more persistent infections.^11^

The inability to treat infections caused by antibiotic-resistant bacteria highlights the need for alternative therapeutic approaches. Among these, nanomaterials are promising alternatives for combating bacterial infections.^13^ Unlike traditional antibiotics, nanoparticles (NPs) may simultaneously target the cell wall and intracellular components via physical interaction,^14^ generation of reactive oxygen species (ROS),^15^ and ion release.^16^ This multifaceted and simultaneous attack poses a significant challenge for bacteria to develop resistance against them.

Nanoparticles (NPs) have been studied as both delivery platforms and antimicrobial agents. Silver nanoparticles (AgNPs) have gathered considerable attention due to their versatile antimicrobial properties and their ability to overcome resistance mechanisms. Various studies have already explored the use of AgNPs with different sizes and chemical composition, either encapsulated within delivery systems or employed directly.^17−25^ However, challenges arise when their application involves cellular interactions, as silver nanoparticles often exhibit high cytotoxicity upon direct contact with mammalian cells.^17,26−30^

Nanoclusters (NCs) represent the next generation of antimicrobials. Silver nanoclusters (AgNCs) exhibit advantageous characteristics compared to nanoparticles, such as a surface layer enriched with silver ions and a higher surface area-to-volume ratio,^31,32^ facilitating an accelerated release of silver ions. Moreover, their small size enables them to effectively penetrate the cellular membrane and interact with microbial components.^33,34^ However, nanoclusters tend to be unstable in physiological conditions.^35^

To address the challenges associated with both nanoparticles and nanoclusters, the utilization of carriers may be a solution. Encapsulation offers the possibility to mitigate the high cytotoxicity levels observed with AgNPs, as it reduces direct contact with cellular membranes. Furthermore, encapsulation can enhance the stability of AgNCs, shielding them from oxidation, aggregation, and loss of antimicrobial activity.

Carriers have previously demonstrated their effectiveness in reducing the adverse effects of both AgNPs and AgNCs.^18,35^ Mesoporous silica nanoparticles (MSN) have shown improved antibacterial activity by preventing the aggregation of silver particles and reducing cytotoxicity. In this study, we investigated the use of polymersomes as carriers for encapsulating AgNPs and AgNCs. Polymersomes are self-assembled vesicles formed by amphiphilic copolymers, known for their excellent stability and biocompatibility with tissues and cells.^36^ Moreover, the surface of polymersomes can be easily and readily functionalized using various chemical handles,^37,38^ making them promising candidates for encapsulating AgNPs and AgNCs and to further compartmentalizing this system for multifunctionality.

## Experimental Methods

### Materials and Instrumentation

During these experiments, analytical-grade chemicals were used. The preparation and characterization of polymers were conducted as described by Rijpkema et al.^39^ Centrifugation steps were performed using an Eppendorf Centrifuge 5430 R. The samples were characterized using various instruments, including the Malvern DLS-ZetaSizer, JEOL JEM-1400 FLASH, Tecan Spark M10 Plate Reader, Wyatt FFF-MALS (Shimadzu HPLC, Damn Heleos-II, Optilab T-rEX, and Eclipse AF4), SP8x AOBS-WLL confocal microscope, and ICP-MS. Data analysis was carried out using the software tools ImageJ and ASTRA 6.1.

### Fabrication of Polymersomes

Polymersomes were prepared following the method previously described by Rijpkema et al.^39^ Briefly, 10 mg of PEG44-PS178 polymer was dissolved in 1 mL of THF and 1,4-dioxane (4:1 v/v) organic solvent. The addition of mQ water (0.5 mL) to the solution was carried out using a flow rate of 1 mL/h, with a time delay of 30 min. The reaction was quenched by adding 6 mL of mQ water, followed by centrifugation at 13,000 rpm for 10 min. Finally, the sample was cleaned with mQ water three times. For the preparation of polymersomes with Nile Red, 25 μL of a 1 mg/mL Nile Red stock solution was added to the mixture.

### Fabrication of Cross-Linked Polymersomes

A modified version of the method previously reported by Rijpkema et al.^39^ was employed. To prepare 70% cross-linked polymersomes, the following solution was prepared under dark conditions: 3 mg of PEG44-PS178, 7 mg of cross-linking polymer PEG44-P(S-*co*-4VBA)150, 20 μL of Irgacure, and 1 mL of THF dioxane (4:1). Subsequently, mQ water (0.5 mL) was added using a flow rate of 1 mL/h, with a 30 min time delay. The solution was degassed by flushing with argon for 3–5 min and then photo-cross-linked with UV light at a wavelength of 450 nm for 5 min at a light intensity of 70. Afterward, the sample was centrifuged at 10000 rpm for 10 min, washed twice with organic solvent THF dioxane (4:1), and finally washed with mQ water. For 100% polymersome cross-linking, the same procedure was followed except for using 10 mg of PEG44-P(S-*co*-4VBA)150 in the initial solution.

### Synthesis of Silver Nanoparticles (AgNP)

Silver nanoparticles were synthesized according to Yan et al.^17^ To produce 20 nm particles, a solution containing 100 mM silver nitrate and 100 mM trisodium citrate (TSC) was prepared with continuous stirring. Subsequently, 6 mL of a 5 mM sodium borohydride solution was added to the mixture. The sample was vigorously stirred for 3 h. Following the stirring process, the sample was centrifuged at 10000 rpm for 10 min and subsequently washed twice with mQ water.

### Synthesis of AgNP in Polymersomes Post-Self-Assembly

If polymersomes were included in the preparation, 100 μL was added simultaneously to the 100 mM silver nitrate and 100 mM TSC solution. Subsequently, the samples were washed with mQ water until the supernatant became clear. The resulting pellet was resuspended in 500 μL of mQ water and transferred to a 220 nm spin filter. The samples were then centrifuged at 14,000 rpm for 10 min and washed with an additional 500 μL of mQ water until no pellet remained. Finally, the particles trapped in the membrane were resuspended in 200 μL of mQ water and stored overnight in the fridge.

### Synthesis of Silver Nanoclusters (AgNC)

AgNCs were synthesized using a modified version of the methodology described by Liu et al.^35^ First, a 112 mM solution of sodium borohydride was prepared by dissolving 4.3 mg of NaBH4 in 200 μL of a 1 M NaOH solution. Subsequently, 800 μL of deionized water was added to the solution. In parallel, a mixture of silver nitrate (50 μL, 20 mM) and GSH (100 μL, 20 mM) was prepared in 1.83 mL of mQ water. Both solutions were combined and vigorously stirred for 1 h. Following that, 20 μL of the 112 mM sodium borohydride solution was added, and the resulting solution was stirred for an additional 5 h. Finally, the sample was centrifuged at 10000 rpm for 10 min.

### Synthesis of AgNC in Polymersomes Post-Self-Assembly

If polymersomes were included in the preparation, 100 μL was added during the mixing of solutions of 112 mM sodium borohydride, 20 mM silver nitrate, and 20 mM GSH.

### Characterization

First, size and surface charge were determined using DLS-Zetasizer. Additionally, transmission electron microscopy (TEM) was employed to visualize all of the samples, providing a visual representation of their structures. For a more detailed characterization, FFF-MALS was utilized, as it can provide further proof and information regarding the positioning of encapsulated AgNP and AgNC. For this purpose, the detector flow rate was set at 1 mL/min, the focus flow rate at 0.5 mL/min, and the inject flow rate at 0.15 mL/min. UV light with a wavelength of 254 nm was utilized. The solvent used during this process was 20 mM NaNO_3_ + 0.02% NaN_3_, and samples were dissolved in mQ water. A regenerative cellulose 10 kDa membrane, obtained from Wyatt, was employed for the FFF-MALS analysis.

### Silver Release Assay

Freshly prepared samples were first normalized using NPN and measured in a plate reader. Subsequently, the samples were centrifuged at 10,000 rpm for 10 min. Following centrifugation, the supernatant was filtered through a 0.22 μm filter and then diluted 1:5 in mQ water. For analysis, a 1:100 dilution of 65% nitric acid was added. The concentration of silver was determined using ICP-MS on days 0, 1, 2, 3, 7, 14, and 30 to reveal the changes in silver concentration over time.

### Antimicrobial Activity Assay

*Pseudomonas aeruginosa* was cultured overnight in Brain Heart Infusion (BHI) broth. Afterward, the cultured *P. aeruginosa* was seeded in μ slides VI 0.5 Glass Bottom (ibidi) and incubated for 3 h. The flow rate was maintained at 0.4 mL/h using BHI media overnight. The study involved the utilization of various samples, including 70-CL, 100-CL, AgNP, 70-CL AgNP, 100-CL AgNP, AgNC, 70-CL AgNC, and 100-CL AgNC. In order to investigate the particle deposition on the biofilm, polymersomes labeled with Nile Red were employed. All samples were normalized using an NPN and measured using a plate reader. Following that, they were injected into the flow at a rate of 0.33 mL/h for 3 h. Subsequently, a LIVE/DEAD assay was performed, omitting the addition of propidium iodide during the staining process for polymersomes labeled with Nile Red. Finally, the stained samples were examined by using confocal microscopy.

### XTT Assay to Assess the Toxicity of Capping Agents

P. aeruginosa cells were grown overnight and diluted the next day 1:100 in 96-well plates to allow for biofilm formation. The biofilms were incubated for 24 h at 37 °C and washed three times with PBS to remove planktonic bacteria, after which 100 mM citrate, 10 mM glutathione, and 20 μg/mL polymyxin B in PBS were added to the wells and left for 3 h to incubate. Wells were washed once again, and XTT (5 μL per well) was added to the wells and left to incubate for another 2 h. Afterward, absorbance was measured at 450 nm using a Tecan Spark M10 plate reader.

### Cell Cytotoxicity Assay

HEK239 cells were cultured in DMEM supplemented with 10% FBS for 3 days. Once the cells reached approximately 60% confluence, they were rinsed three times with 1x PBS, pH 7.4, and detached using 4 mL of trypsin for 3 min. Trypsin was quenched by adding 8 mL of DMEM. The cells were then transferred to a 15 mL Falcon tube and centrifuged at 0.3 rcf for 5 min. After discarding the supernatant, the cells were seeded in a 96-well plate with DMEM complete medium at a density of 5 × 10^5^ cells/ml and incubated for 24 h at 37 °C with 5% CO_2_. Next, different concentrations of treatments in DMEM complete medium were added to the wells and incubated at 37 °C with 5% CO_2_ for either 24 or 48 h. Following the incubation period, 10 μL of CCK8 (Sigma-Aldrich) was added to each well and incubated for 3 h. The absorbance was then measured at 450 nm.

## Results and Discussion

**Figure 1:**
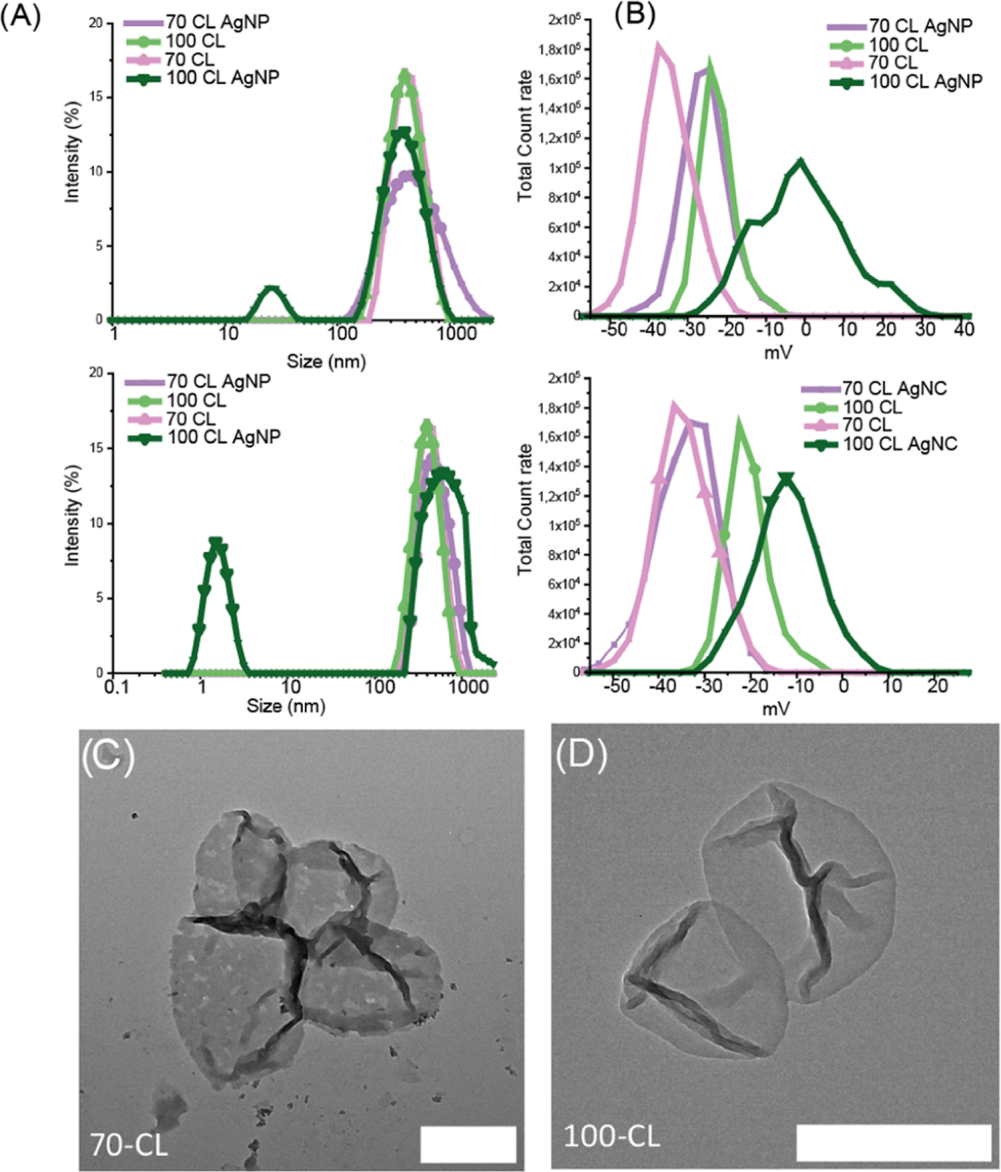
Characterization of empty and fully assembled systems. (A) Size distribution and (B) surface charge of AgNP and AgNC systems as determined by DLS-Zetasizer. TEM images of 70-CL (C) and 100-CL (D).

Cross-linked polymersomes were prepared using the method previously described by Rijpkema et al.^39^ AgNPs and AgNCs were synthesized according to Yan et al.^17^ and Liu et al.^35^ and checked for size, surface charge, UV–vis, and TEM (S1). To investigate the impact of encapsulation in polymersomes, two samples were prepared, namely, 70 and 100% cross-linked polymersomes. Both samples exhibited uniform distribution and displayed a peak at approximately 400 nm in DLS-Zetasizer (Figures 1A,B and S2), which is consistent with the employed method.^39^ To determine the pore size of the 70% cross-linked polymersomes, TEM images were analyzed using ImageJ (Figure 1C). The average pore size was 28 nm (±9 nm), which aligns with earlier studies.^39^ On the other hand, no visible pores were observed on the surface of the 100% cross-linked polymersomes (Figure 1D), indicating that neither AgNPs nor AgNCs would be able to penetrate the core and be encapsulated.

The fully assembled systems, composed of porous 70-CL AgNP or AgNCs and 100-CL AgNP and AgNCs, were subjected to size and surface charge analysis (Figure 1A). The porous polymersomes 70-CL showed peaks at around 400 nm, aligning with the observed size for empty 70-CL polymersomes.^39^ 100-CL AgNC shifted in size to around 660 nm, while 100-CL AgNP displayed another peak at around 25 nm. This indicates that nanoclusters have formed on the outer side of the polymersomes, sticking to the membrane and even “breaking off” and going back into solution in the case of 100-CL AgNP.

The surface charge of polymersomes can provide more information on particles accumulating around the shells or on the inside of carriers due to shifts in zeta potential. Silver ions and particles on the outer shell increased the surface charge of nonporous polymersomes (Figure 1B), while porous polymersomes retained a charge close to that of their empty counterpart. These data suggest that AgNCs and AgNPs were successfully formed in the inner compartment of porous polymersomes.

**Figure 2:**
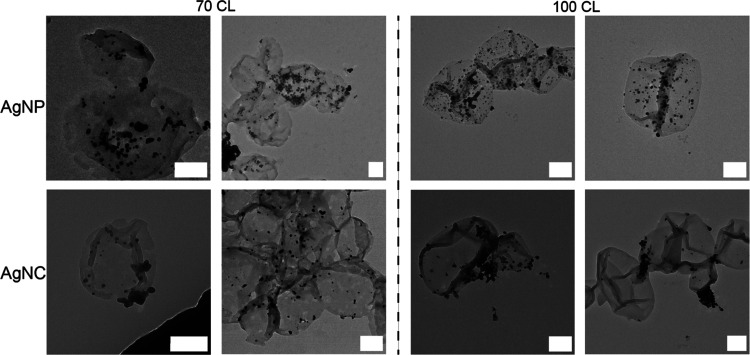
TEM analysis of systems composed of 70-CL (left) and 100-CL (right) polymersomes containing AgNPs (top row) or AgNCs (bottom row). Scale bar represents 200 nm.

To further study the morphology of the fully assembled system, we employed TEM (Figure 2). AgNPs and AgNCs can be seen covering both 70-CL and 100-CL polymersomes with clustering of AgNCs on the surface of 100-CL. Determining the inner or outer location of silver is difficult with this technique, which is why additionally FFF and total silver content serve as proof of compartmentalized and noncompartmentalized polymersomes.

**Figure 3:**
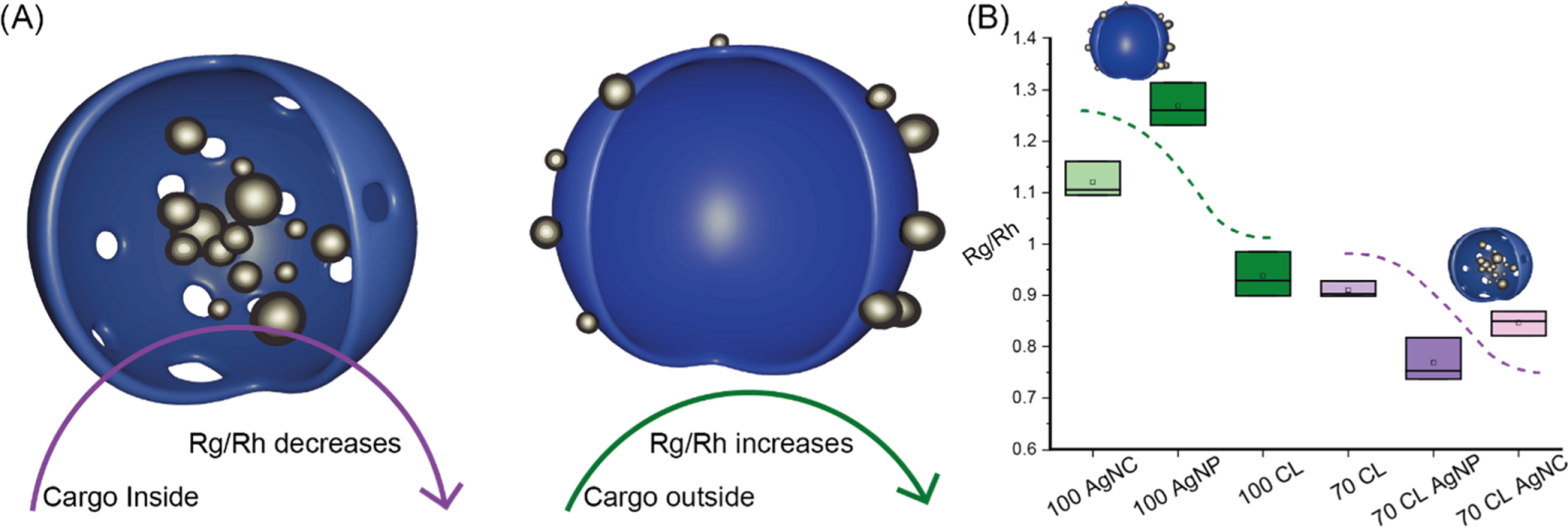
FFF-MALS characterization of the cargo location. (A) Overview of *R*_g_/*R*_h_ value response to cargo inside or outside of the polymersomes as can be seen in experimental data (B).

Center of mass, or radius of gyration (*R*_g_)/hydrodynamic radius (*R*_h_), has been previously used to determine the loading of carriers with various cargo following the general idea of increased *R*_g_/*R*_h_ when loaded (Figure 3A).^40−45^ Spherical particles will have the *R*_g_ located at the surface of the particle, matching the *R*_h_, causing the center of mass to equal 1.^43^ When *R*_g_ becomes lower due to loading of cargo in the center of a polymersomes, the center of mass will become smaller.^44^ Due to the nucleation of silver nanoparticles and nanoclusters in the core of 70-CL, a decrease in *R*_g_/*R*_h_ is observed (Figures 3B and S3), whereas the increase of mass on the outer surface of 100-CL increases the *R*_g_/*R*_h_ compared to the native particles (Figures 3B and S3). Conclusive evidence of a compartmentalized system containing silver cargo is therefore given.

**Figure 4:**
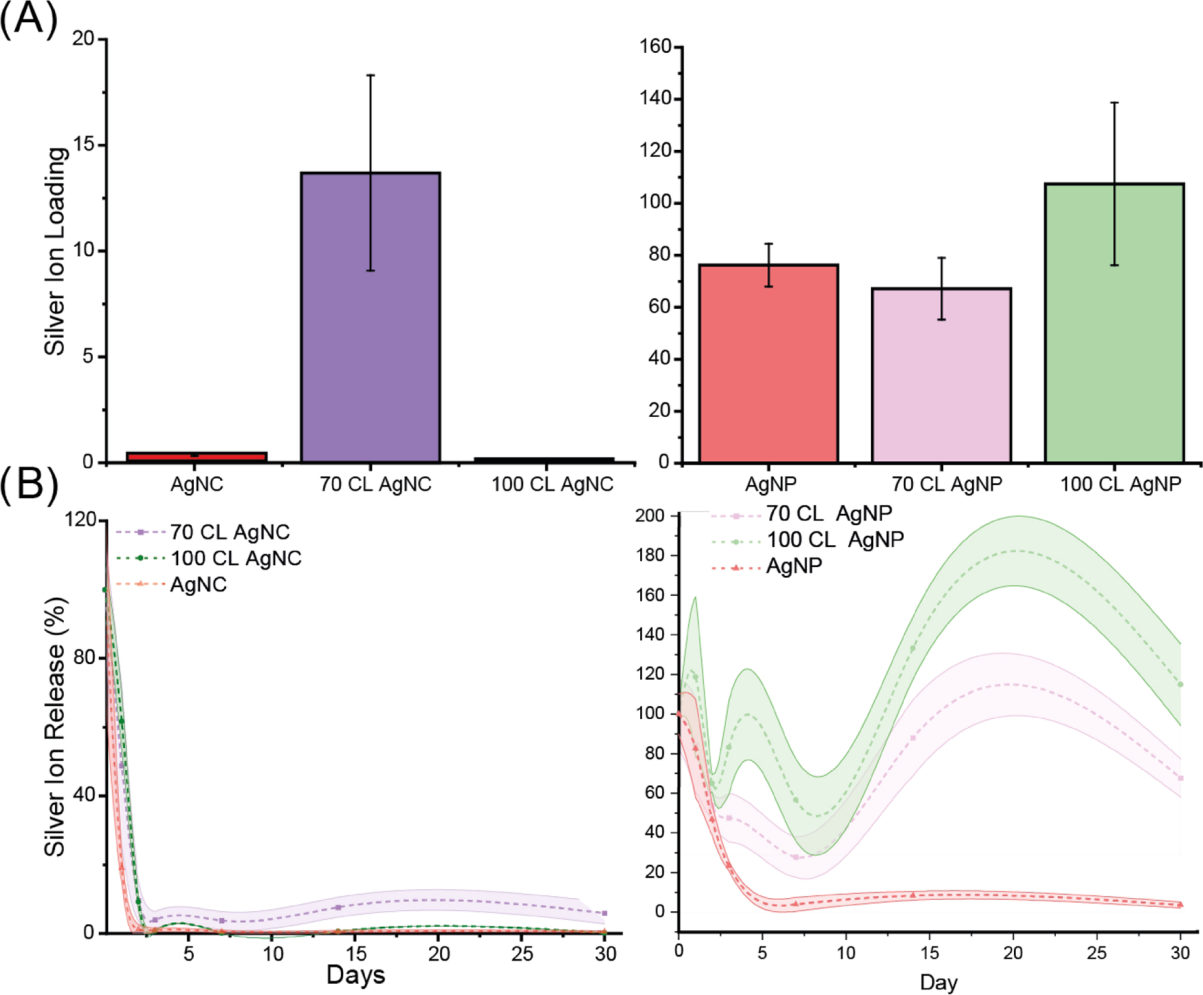
ICP-MS analysis of total silver content in AgNP and AgNC systems (A) and silver ion release over 30 days in % relative to day 0 (B).

The total silver content of the different systems was measured to showcase different silver retention capabilities (Figure 4A). 70-CL AgNC showed a significantly higher Ag^+^ ion count compared to 100-CL and pure AgNCs, indicating increased nucleation in the core as well as retaining formed particles. Contents of AgNP system show no statistically significant differences in Ag levels, exposing a failed retainment of silver from 70-CL polymersomes. To investigate the potential of polymersomes in enhancing the stability of silver particles and clusters, an ion release assay was conducted (Figure 4B). Remarkably, both 70-CL and 100-CL polymersomes loaded with AgNPs demonstrated sustained stability throughout the experiment (Figures 4B and S4), while the ion content of AgNCs exhibited a rapid decrease at the onset of the experiment. Previous studies have shown a substantial decline in AgNPs within the initial 72 h period, followed by a stabilization phase.^17^ Notably, the presence of both 70-CL and 100-CL polymersomes contributed to the improved stability of the silver nanoparticles. We propose that the protective nature of polymersomes, likely through encapsulation, may account for this effect. We observe though that nucleation at the membrane of 100-CL polymersomes and subsequent attachment are followed by slower disintegration of AgNPs. We hypothesize that interaction with the polymersome membrane leaves less surface area of AgNPs to interact with the harsh dissolving environment, resulting in prolonged ion release.

Silver nanocluster systems displayed a massive reduction of ions due to their unstable nature within the first 3 days, for both 100-CL AgNCs and AgNCs, a substantial reduction of 140-fold and 105-fold, respectively. On the contrary, the release of silver ions from 70-CL AgNCs remains relatively stable over time. Notably, the silver release from 70-CL AgNCs is significantly higher compared to that from both AgNCs and 100-CL AgNCs after 14 and 30 days. These findings highlight the unique capability of 70-CL polymersomes in preventing the rapid oxidation of silver within nanoclusters by utilizing the core as a protective compartment and allowing for the sustained release of ions for antimicrobial purposes.

**Figure 5:**
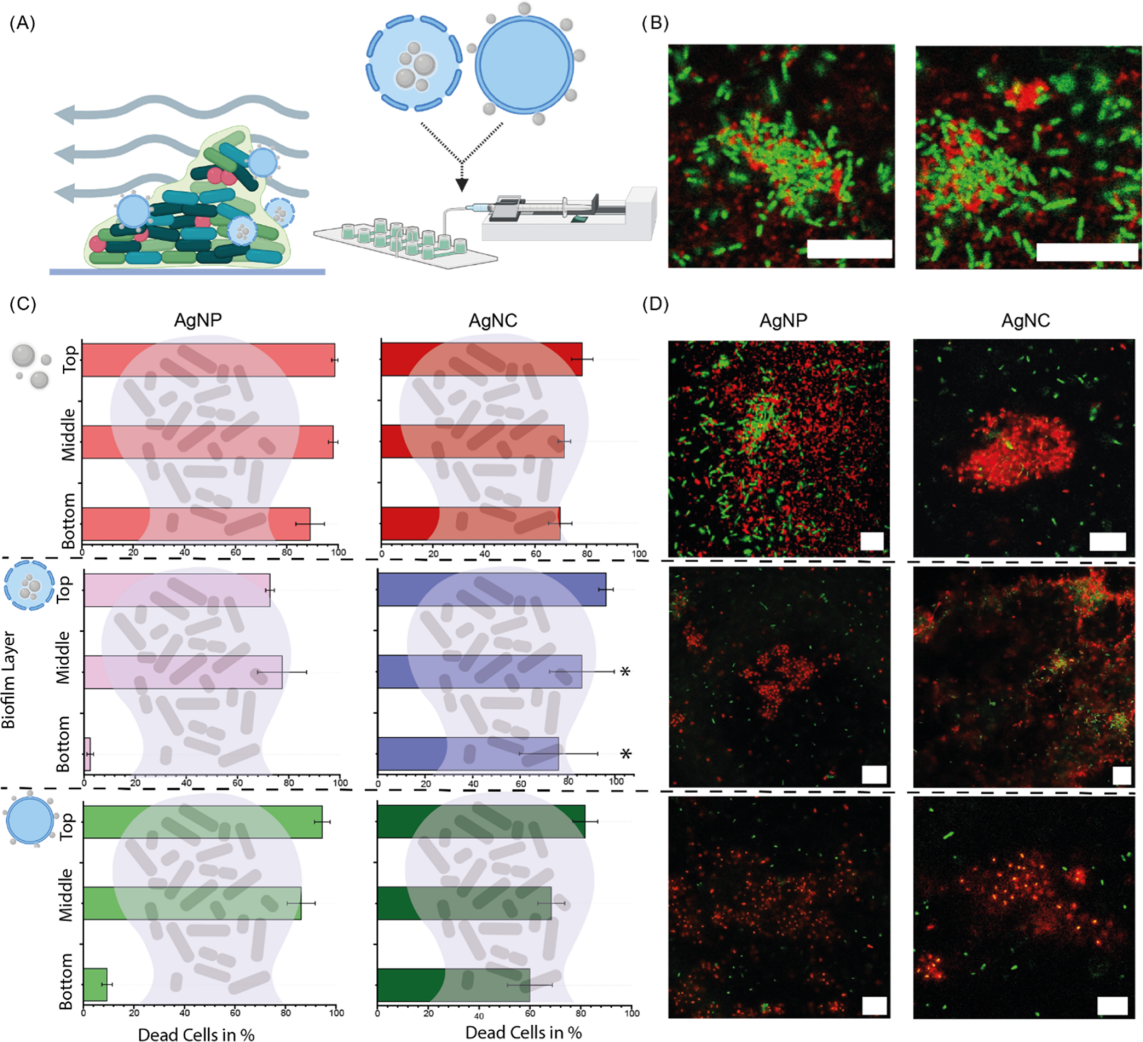
Antimicrobial activity of all systems in *P. aeruginosa*. (A) Overview of the experimental setup with flow. (B) Accumulation of empty polymersomes (red channel) in biofilms (green channel) under flow. (C) Quantified viability across top, middle, and bottom biofilm layers, derived from confocal images (D).

Effects of empty polymersomes were studied under flow conditions (Figure 5A) to confirm adherence to the biofilm (Figure 5B) and to rule out cytotoxic effects from only polymersomes (Figure S5). Antimicrobial activity assays were performed to study the effects of polymersomes with AgNPs and AgNCs on *P. aeruginosa* biofilms in a flow environment, mimicking a more natural as well as challenging environment for antimicrobials (Figures 5C,D and S6). After exposure, the bacteria in the biofilms were analyzed for their cell wall integrity utilizing confocal laser scanning microscopy, indicating the viability of bacteria throughout the biofilms. To further gain insight into the effectiveness of the systems, the analysis of single biofilms was split into bottom, middle, and top layers of the biofilm. All samples showed high antimicrobial activity on the top layers, yet different effectiveness was observed for the middle and bottom layers (Figure 5C).

The efficacy of AgNPs on the bottom layer was found to be ineffective for both 70-CL and 100-CL systems, with eradication rates of only 2.45% ± 1.3 and 9.38% ± 2.04, respectively. Conversely, AgNCs, including both 70-CL and 100-CL, demonstrated notable eradication of the bottom layer with rates of 76.22% ± 16.51 and 59.98% ± 8.8, respectively. Interestingly, there were no significant differences observed in the efficacy on the bottom layer between 70-CL AgNCs and pure AgNCs, whereas 100-CL AgNC exhibited a significantly lower efficiency in eradicating the bottom layer as well as the middle layer, putting the compartmentalized system on the same antimicrobial level as pure AgNCs. To rule out the effects of the different capping agents on the antimicrobial effects, biofilms were exposed to citrate, glutathione in the concentrations used for silver particle synthesis (Figure S7). No effects were observed when compared to the negative control (PBS), unlike the positive control Polymyxin B, a broad-range antibiotic, which showed a significant decrease in viability. Antimicrobial effects observed by fully assembled systems are therefore to be attributed to released silver, not the capping agents.

The great challenge in combating biofilms has been disrupting the bottom layer, where often dormant cells are found. Ideally, particles should exhibit high antimicrobial activity across all three layers, namely, the top, middle, and bottom. While the addition of a carrier hinders this activity in the case of AgNPs, 70-CL AgNCs do not impede antimicrobial efficacy, potentially due to sustained release of ions and prolonged protection of the AgNCs in the core of polymersomes, whereas 100-CL AgNCs suffer from immediate dissolution.

**Figure 6:**
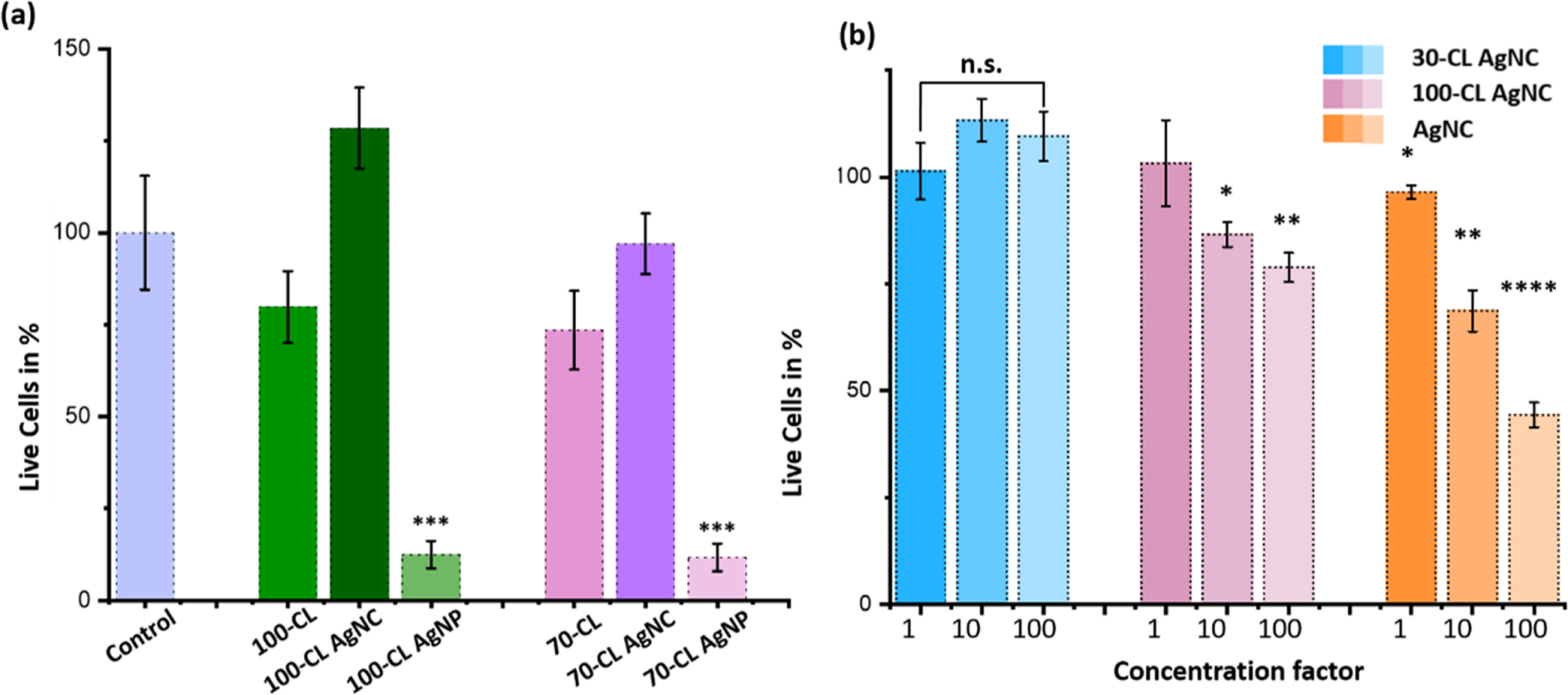
Cell cytotoxicity. (a) After treatment with 70-CL AgNP, 100-CL AgNP, 70-CL AgNC, and 100-CL AgNC. (b) After treatment with different concentrations of AgNC, 70-CL AgNC, and 100-CL AgNC. Experiments were carried out in replicates of 5–10, Unpaired *t* test with *p* ≤ 0.05 was conducted to determine statistical significance.

An optimal system is expected to have high cytocompatibility, so high doses can be administered without fear of damaging healthy cell tissue. Both 70-CL and 100-CL AgNPs significantly decreased the cell viability. On the other hand, no significant differences were found between 70-CL and 100-CL AgNCs, and the control (Figures 6a and S8). To further investigate, concentrations of AgNC systems used for antimicrobial assays were increased 10- and 100-fold. While pure AgNCs and 100-CL AgNCs quickly deteriorate the viability of cells, 70-CL AgNC retained excellent cytocompatibility (Figure 6b).

Previous studies have consistently demonstrated the adverse effects of silver particles and ions on various systems within the human body, such as the respiratory, digestive, and reproductive systems.^30,46^ Nevertheless, the application of silver nanoparticles has shown promise in mitigating cell cytotoxicity due to their protective outer layer.^47^ However, the presence of citrate hinders cell viability in these nanoparticles.^48^ In contrast, silver nanoclusters possess an outer layer composed of glutathione, a naturally occurring nontoxic compound in the human body. Remarkably, recent research has illustrated that the cell viability of silver nanoclusters is 4 times higher compared to silver ions, further highlighting their potential as a safer alternative.^35^

To further investigate the effect of AgNCs on cell cytotoxicity, the concentrations of AgNCs, 100-CL AgNCs, and 70-CL AgNCs were increased by 10- and 100-fold (Figure 6b). Notably, even a 10-fold increase in the concentration of AgNCs resulted in significant cytotoxicity. Moreover, when the concentration of 100-CL AgNCs was increased by 10-fold, clear indications of cytotoxicity were observed. Surprisingly, in the case of 70-CL AgNCs, even a 100-fold increase in concentration did not lead to an increase in cell cytotoxicity. These findings provide robust evidence that 70-CL AgNCs are nontoxic to human cells, even at concentrations 100 times higher than those previously employed for antimicrobial purposes. Therefore, higher doses of 70-CL AgNCs can be utilized to enhance antibacterial activity without compromising cell cytotoxicity. We hypothesize that the reduced contact of AgNC with the cell membranes decreases the cytotoxicity observed, whereas systems with direct contact led to significant damage.

Antibiotic resistance has rendered traditional antibiotics obsolete, highlighting the development of new strategies to effectively combat infections caused by these microorganisms. This research focuses on the utilization of encapsulated silver nanoparticles and nanoclusters as potential antimicrobial agents. The high antimicrobial activity coupled with minimal cytotoxicity exhibited by encapsulated silver nanoclusters makes them highly promising for antimicrobial treatments. However, it is worth noting that these nanoclusters exhibit low stability even when encapsulated. Therefore, it is imperative to dedicate further efforts toward optimizing silver nanoclusters, aiming to preserve their remarkable antimicrobial activity and low cytotoxicity while enhancing their stability. Considering the trend toward a selectivity of 70-CL AgNC, sparing human cells and exhibiting antimicrobial activities, we foresee this system as having great potential in the medical field, i.e., as topical antibiofilm compound after a surgical site infection.

## Conclusions

To summarize, we developed various systems utilizing silver nanoparticles or silver nanoclusters as cargo, either on the surface of cross-linked polymersomes or formed in the core of porous polymersomes, resulting in an ion-releasing compartment. In-depth characterization was used to verify the localization of particles and clusters on and inside the polymersomes. Ion-releasing assays confirmed the hypothesis that entrapping particles in the core enhances stability, which in turn significantly increases the antimicrobial effectiveness while allowing the system to have good mammalian cell compatibility. These findings establish the promising prospects of 70-CL AgNCs as an effective and safe antimicrobial treatment, offering a valuable alternative to combat antibiotic resistance by the compartmentalization of toxic nanoclusters inside porous polymersomes.

## Data Availability

All of the data supporting the conclusion are available in the paper and/or the Supporting Information.

## Supporting Information Available

Characterization of AgNCs and AgNPs, FFF-MALS data, ion release, biofilm viability when exposed to empty polymersomes, biofilm viability when exposed to capping agents, and cell viability (PDF)
