# Supplementary material for: Porous Polymersomes as Carriers for Silver Nanoparticles and Nanoclusters: Advantages of Compartmentalization for Antimicrobial Usage
Source: Biomacromolecules. 2023 Nov 10;24(12):5905–14. doi: 10.1021/acs.biomac.3c00925 (PMC10716846; doi:10.1021/acs.biomac.3c00925)
Supplement: Supplementary file 1 — bm3c00925_si_001.pdf [file bm3c00925_si_001.pdf]

Porous Polymersomes as Carriers for Silver Nanoparticles and Nanoclusters: Advantages of Compartmentalization for Antimicrobial Usage

*Bela B. Berking[1], Lucía Mallen-Huertas[1], Sjoerd J. Rijpkema, Daniela A. Wilson**

Systems Chemistry Department, Institute for Molecules and Materials, Radboud University, Nijmegen, The Netherlands

Table of Contents

1. Characterization 3
2. Ion Release via ICP-MS 5
3. Antimicrobial Analysis 5
4. Cell Cytotoxicity 7

Supplementary Information

1. Characterization

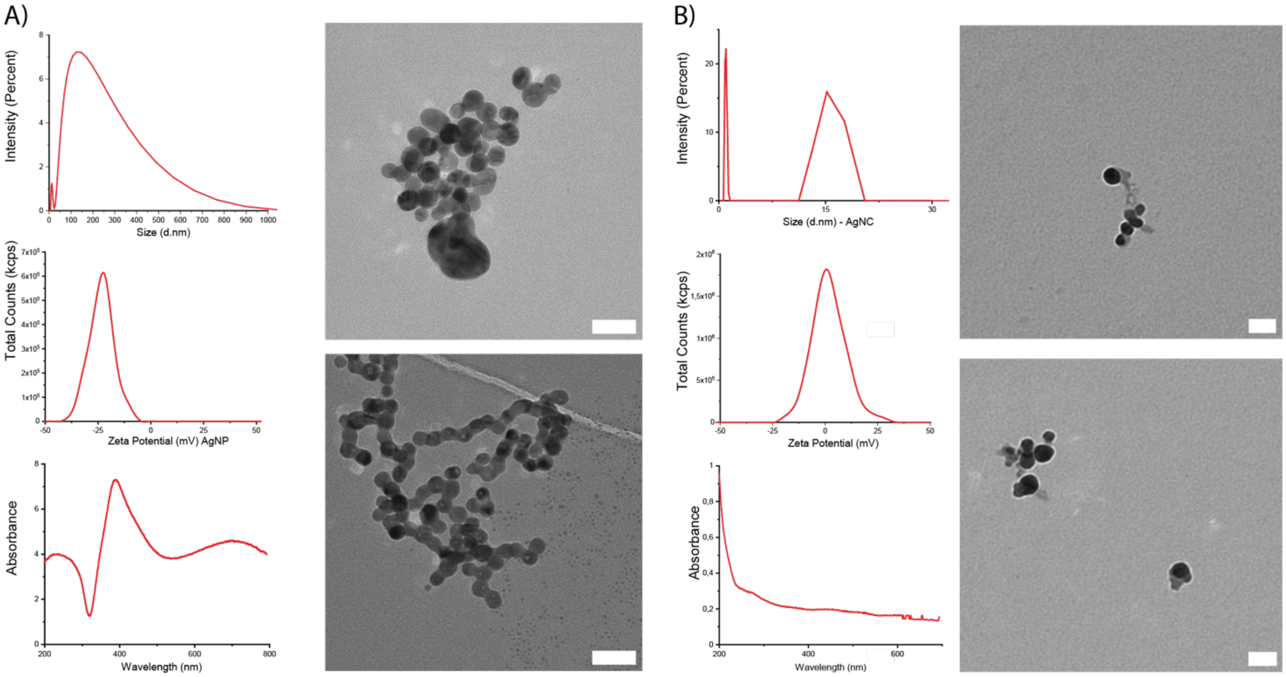

Figure S1. AgNP (A) and AgNC (B) Characterization comprised of DLS, Zeta potential, UV VIS, and TEM. Scale bars set at 50 nm

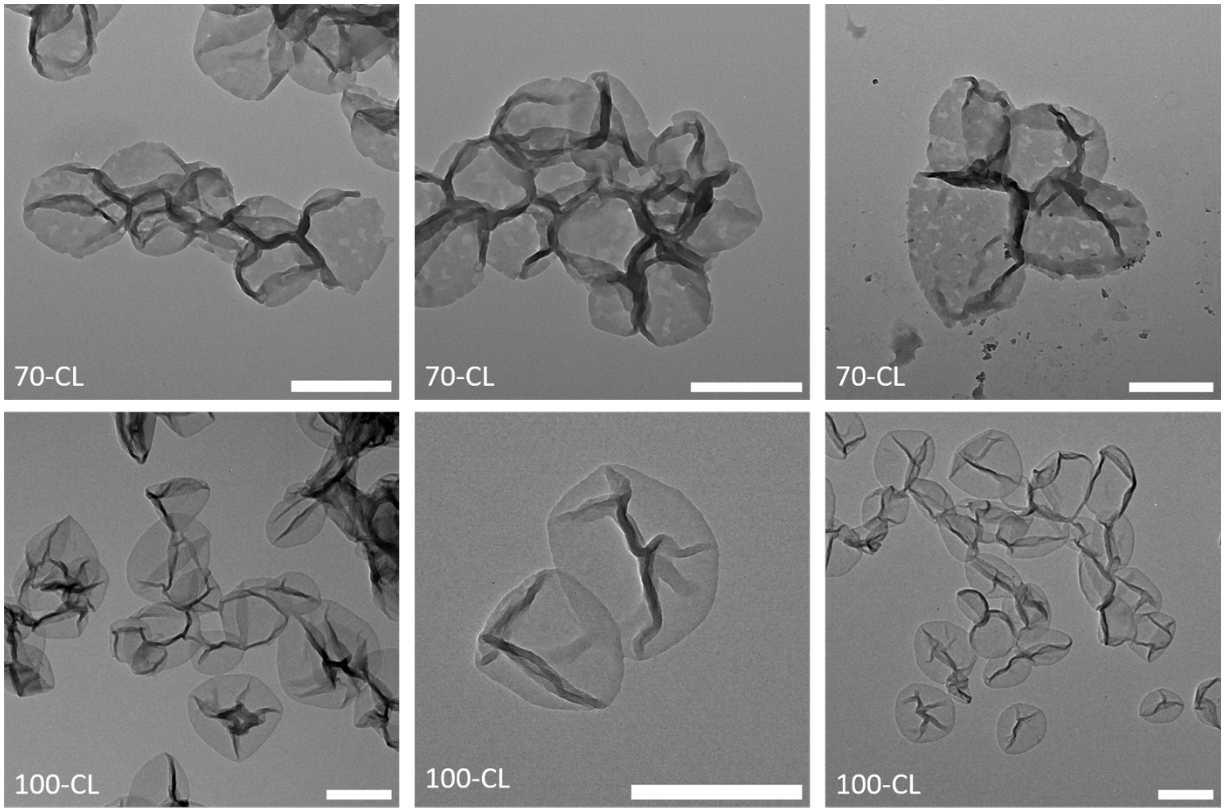

Figure S2. TEM Images of 70-CL and 100-CL Polymersomes. Scale bar 500 nm.

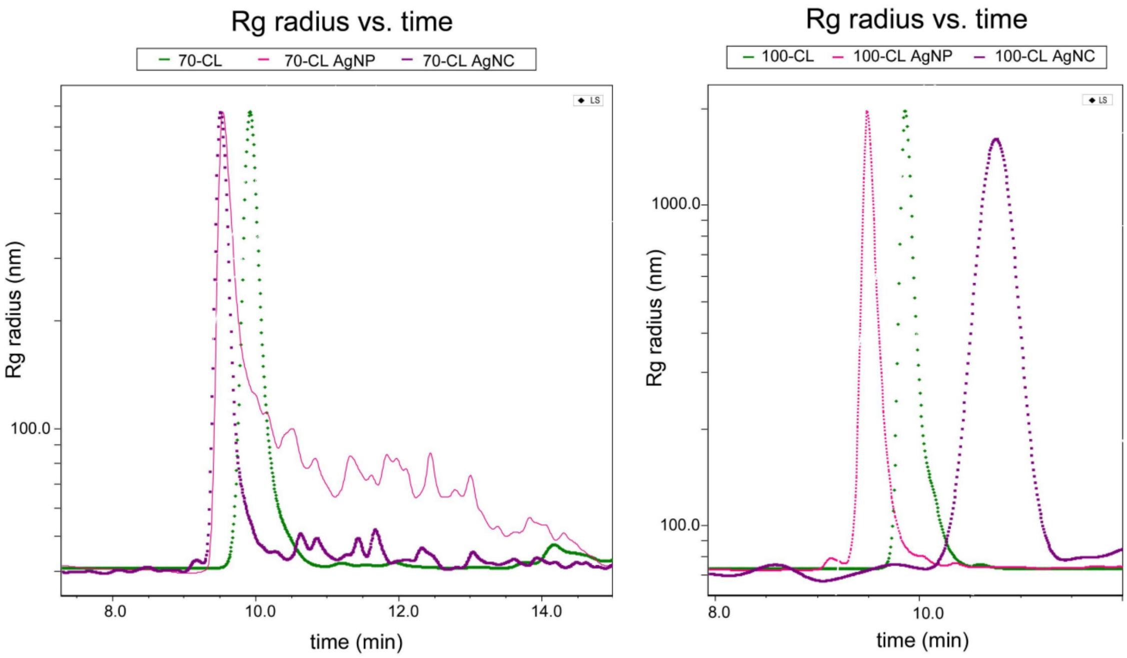

Figure S3. Rg radius (nm) obtained by FFF-MALS

2. Silver Ion Release via ICP-MS

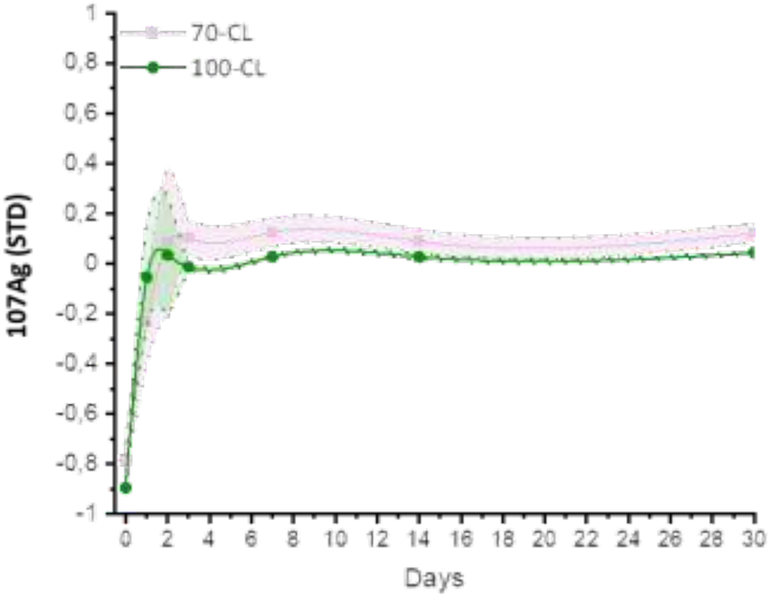

Figure S4. Silver release of empty controls 70-CL and 100-CL.

3. Antimicrobial Analysis

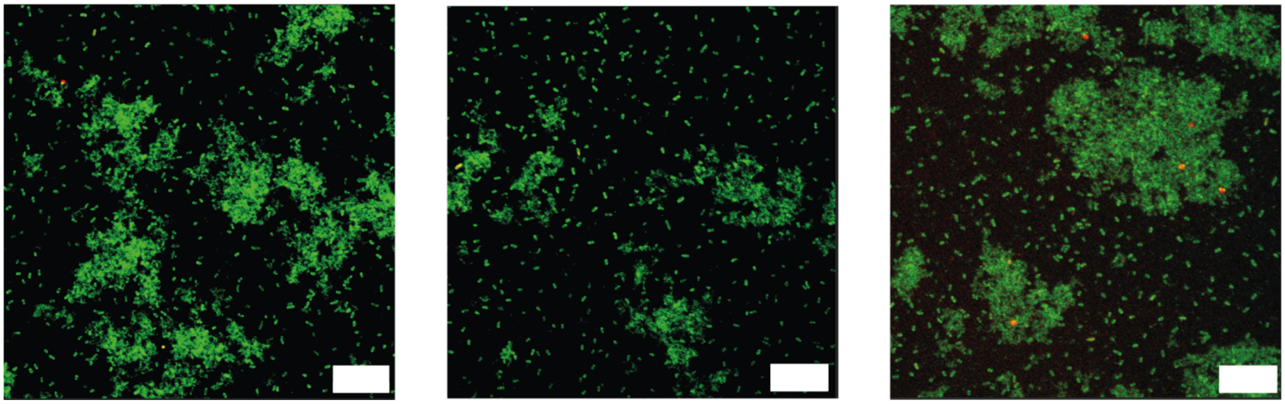

Figure S5. Effect of empty polymersomes on biofilm viability using Live/Dead Assay. Syto9 (Green Channel) representing viable cells, while PI stained cells are non-viable. Scale bar set to 10 µm.

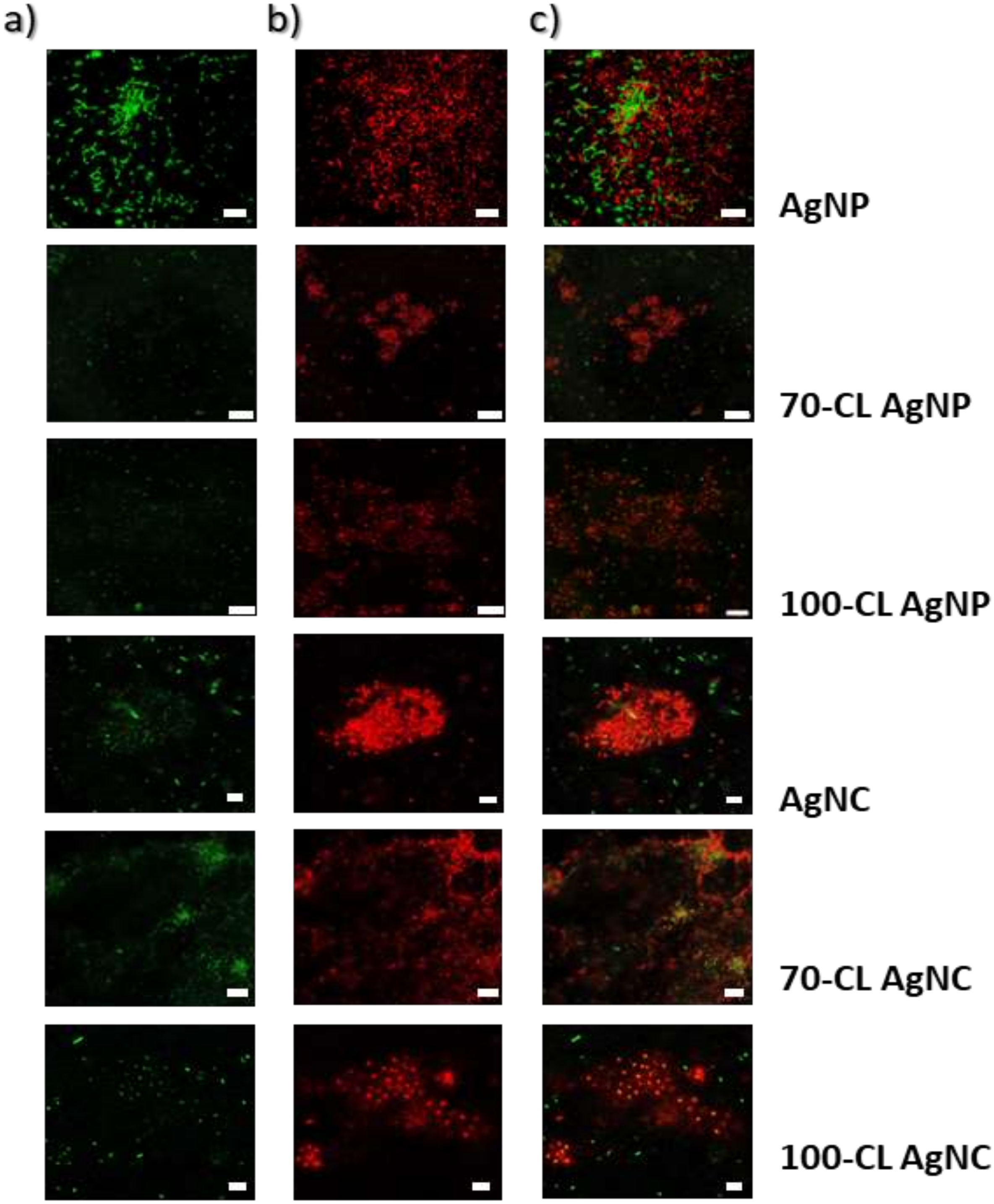

Figure S6. Confocal images of *P. aeruginosa* biofilms showing (a) live, (b) dead, and (c) merged in response to treatment with AgNPs, 70-CL AgNP, 100-CL AgNP, AgNCs, 70-CL AgNC, and 100-CL AgNC. Scale bar 15 μm.

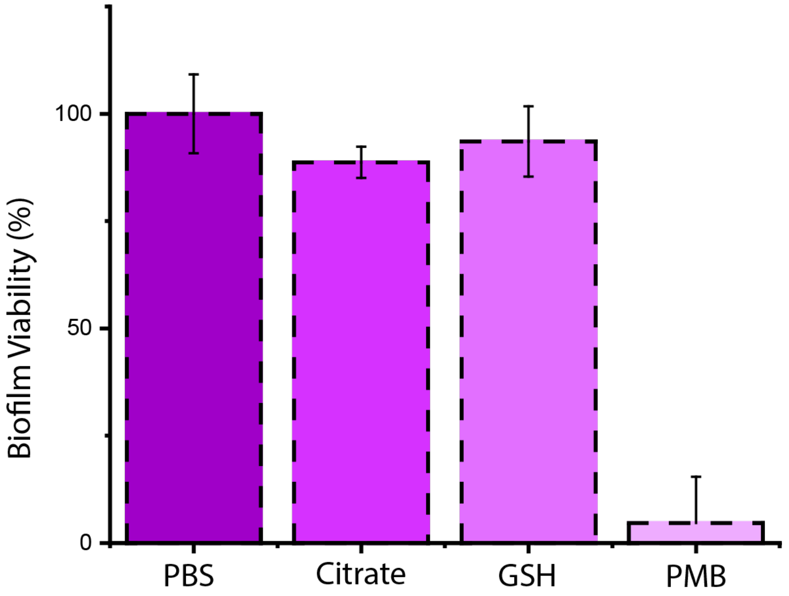

Figure S7. Biofilm viability assay using PBS, Citrate, Glutathione (GSH), and Polymyxin B (PMB)

4. Cell Cytotoxicity

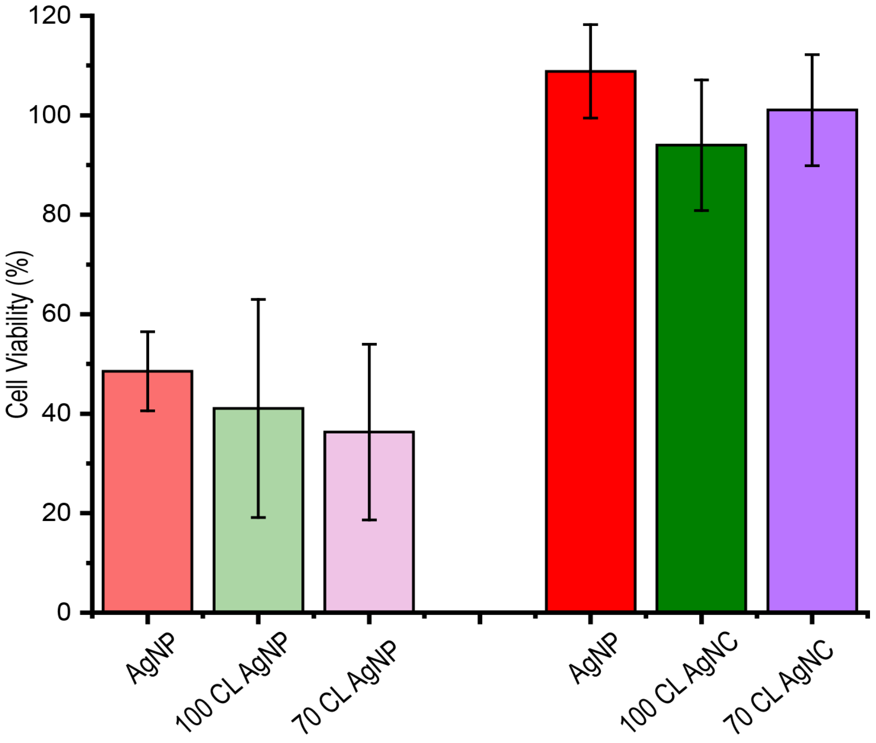

Figure S8. Repetition of cytotoxicity assay.
